# Associação de Injúria Miocárdica e Mortalidade em Pacientes Hospitalizados com COVID-19

**DOI:** 10.36660/abc.20201284

**Published:** 2021-02-19

**Authors:** Edson Romano

**Affiliations:** 1 Hospital do CoraçãoUnidade de Terapia IntensivaSão PauloSPBrasilHospital do Coração - Unidade de Terapia Intensiva / Cardiologia,São Paulo, SP – Brasil

**Keywords:** COVID-19, Coronavírus, Betacoronavírus, SARS CoV-2, Infecção, Miocardite, Infarto do Miocárdio, Hospitalização, Morbidade

A doença Covid-19 surgiu em dezembro de 2019 na cidade de Wuhan, província de Hubei, China, e tem como causa o novo coronavírus denominado *severe acute respiratory syndrome coronavirus 2* (SARS-CoV-2), sétimo coronavírus identificado até o momento, diferente dos outros coronavírus que causam pneumonia e resfriado comum.^1^Devido à maior e mais rápida transmissibilidade, foi declarada uma pandemia pela Organização Mundial de Saúde, em 11 de março de 2020.^2^

Desde então, vários estudos a nível mundial têm sido realizados e publicados com o intuito de determinar os fatores de risco para desenvolver a doença, bem como suas complicações, graus de gravidade, tratamento e morbi-mortalidade.

Um dos estudos iniciais foi realizado pelo Centro Chinês para Controle e Prevenção de Doenças que avaliou o grau de gravidade de 72.314 pacientes com Covid-19 nessa população. Em 81,4% dos casos a doença foi classificada como leve, grave em 13,9% e crítica em 4,7%.^3^

Quanto ao acometimento cardíaco, a manifestação se dá por injúria miocárdica, que é definida pelo nível elevado de troponina e que ocorre especialmente devido a processos miocárdicos não isquêmicos, incluindo infecção respiratória grave com hipoxia, sepse, inflamação sistêmica, tromboembolismo pulmonar, hiperestimulação adrenérgica cardíaca durante a síndrome da tempestade de citocinas e possivelmente miocardite por ação direta do vírus. A etiologia isquêmica também se faz presente por ruptura de placa aterosclerótica coronariana, espasmo coronário, microtrombos ou lesão endotelial direta.^4^

Um aspecto significativo, que tem sido descrito, é que pacientes que apresentam injúria miocárdica estão associados a maior necessidade de suporte ventilatório e mortalidade intra-hospitalar. São pacientes geralmente mais idosos, com maior prevalência de hipertensão arterial sistêmica, diabetes mellitus, doença arterial coronariana e insuficiência cardíaca.^5^

Em uma revisão sistemática de 4 estudos com 374 pacientes, os níveis de troponina I foram significativamente maiores nos pacientes com Covid-19 grave, em comparação com a doença não grave.^6^

Quanto à prevalência, estudos desenvolvidos na China têm relatado injúria miocárdica com níveis elevado de troponina variando de 7% a 17% em pacientes hospitalizados, de 22% a 31% entre pacientes admitidos nas unidades de terapia intensiva (UTI) e de até 59% dos doentes que evoluíram a óbito.^7,8^ Essa variação na incidência de lesão miocárdica tem sido replicada em várias publicações por diferentes centros, como também demonstrada por uma meta-análise de 10 estudos envolvendo 3.118 pacientes em Wuhan, China, com prevalência de injúria miocárdica variando de 15% a 44% e o efeito combinado desses estudos mostrou que pacientes com troponina elevada tiveram risco de mortalidade 21 vezes maior (OR = 21,15).^9^Essa variação reflete a heterogeneidade das definições de injúria miocárdica da população estudada e das características regionais, o que de certa forma também vai refletir nas taxas de letalidade, com dados mostrando incidência de 13% a 67% de pacientes com troponina elevada internados na UTI.^9^

Faz-se, portanto, necessário um estudo brasileiro para avaliação da presença de injúria miocárdica na nossa população, no que diz respeito à mortalidade desse grupo de pacientes, bem como à presença de comorbidades como preditor de óbito.

Nesta edição, autores brasileiros^10^ apresentam o resultado de um estudo observacional, retrospectivo, realizado na UTI de um hospital particular no Rio de Janeiro, entre março e abril de 2020. Foram incluídos inicialmente 105 casos confirmados de Covid-19. Após exclusão de 35 pacientes por ausência de dosagem de troponina I e 9 por insuficiência renal grave, restaram 61 pacientes e destes 36% evoluíram com injúria miocárdica, ou seja, troponina I elevada. O objetivo primário foi o de descrever a incidência de injúria miocárdica e identificar variáveis associadas à sua ocorrência. O objetivo secundário foi o de avaliar a troponina I ultrassensível como preditor de mortalidade hospitalar.

Após análise univariada e regressão logística multivariada de uma série de variáveis gerais e comorbidades, os preditores de injúria miocárdica com significância estatística foram a hipertensão arterial sistêmica e o índice de massa corpórea. A taxa de mortalidade do grupo foi de 24,6%, sendo que o valor da troponina I teve relação com a mortalidade hospitalar.

Apesar deste estudo ter limitações importantes, como o pequeno número de pacientes e a perda de casos por ausência de dosagem de troponina I, ficou demonstrado o impacto da injúria miocárdica na mortalidade hospitalar e a identificação de preditores de risco como a hipertensão arterial sistêmica e o índice de massa corpórea.

Estudos nacionais mais robustos e multicêntricos] poderão confirmar os achados revelados neste estudo, bem como traçar um perfil da população brasileira.
