# Supplementary material for: Associação de Injúria Miocárdica e Mortalidade em Pacientes Hospitalizados com COVID-19
Source: Arq Bras Cardiol. 2021 Feb 19;116(2):283–4. [Article in Portuguese] doi: 10.36660/abc.20201284 (PMC7909984; doi:10.36660/abc.20201284)
Supplement: Translation

# Translation: Association of Cardiac Injury with Mortality in Hospitalized Patients with COVID-19

**Authors:** Edson Romano

**Affiliations:** 1 BrazilHospital do Coração - Unidade de Terapia Intensiva / Cardiologia, São Paulo, SP – Brazil

**Keywords:** COVID-19, Coronavirus, Betacoronavirus, SARS CoV-2, Infection, Myocarditis, Myocardial Infarction, Hospitalization, Morbidity

Covid-19 disease emerged in December 2019 in Wuhan City, Hubei Province, China. It is caused by the new coronavirus called severe acute respiratory syndrome coronavirus 2 (SARS-CoV-2), the seventh coronavirus identified to date, which is different from other coronaviruses that cause pneumonia and common cold.^1^Due to the greater and faster transmissibility, a pandemic was declared by the World Health Organization on 11 March 2020.^2^

Since then, several studies worldwide have been conducted and published with the aim of determining the risk factors for developing the disease, as well as its complications, degrees of severity, treatment, morbidity, and mortality.

One of the initial studies was conducted by the Chinese Center for Disease Control and Prevention, which evaluated the degree of severity of 72,314 patients with Covid-19 in this population. In 81.4% of the cases, the disease was classified as mild, severe in 13.9%, and critical in 4.7%.^3^

Regarding cardiac involvement, the manifestation occurs with myocardial injury, which is defined by high levels of troponin, and it occurs especially due to non-ischemic myocardial processes, including respiratory infection with severe hypoxia, sepsis, systemic inflammation, pulmonary thromboembolism, cardiac adrenergic hyperstimulation during cytokine storm syndrome, and possibly myocarditis due to direct action of the virus. Ischemic etiology is also present due to rupture of coronary atherosclerotic plaque, coronary spasm, microthrombi, or direct endothelial lesion.^4^

A significant aspect that has been described is that patients with myocardial injury are associated with a greater need for ventilatory support and in-hospital mortality. They are generally older patients with a higher prevalence of systemic arterial hypertension, diabetes mellitus, coronary artery disease, and heart failure.^5^

In a systematic review of 4 studies with 374 patients, troponin I levels were significantly higher in patients with severe Covid-19 compared to those with non-severe forms of the disease.^6^

Regarding prevalence, studies developed in China have reported myocardial injury with elevated troponin levels ranging from 7% to 17% in hospitalized patients, from 22% to 31% among patients admitted to intensive care units (ICU), and up to 59% of patients who died.^7,8^ This variation in the incidence of myocardial injury has been replicated in various publications in different centers, and it has also been demonstrated in a meta-analysis of 10 studies involving 3,118 patients in Wuhan, China, with a prevalence of myocardial injury ranging from 15% to 44%; the combined effect of these studies showed that, in patients with high troponin, the mortality risk was 21 times higher (OR = 21.15).^9^This variation reflects the heterogeneity of the definitions of myocardial injury and of the population studied and regional characteristics, which will in some way also reflect the lethality rates, with data showing an incidence of 13% to 67% of patients with elevated troponin hospitalized in the ICU.^9^

Therefore, a Brazilian study is necessary to evaluate the presence of myocardial injury in our population, with regard to the mortality of this group of patients, as well as the presence of comorbidities as a predictor of death.

In this edition, Brazilian^10^ authors present the results of an observational, retrospective study conducted in the ICU of a private hospital in Rio de Janeiro, between March and April 2020. Initially, 105 confirmed cases of Covid-19 were included. After excluding 35 patients due to the absence of troponin I and 9 due to severe renal failure, 61 patients remained, and 36% of them presented myocardial injury with elevated troponin I. The primary objective was to describe the incidence of myocardial injury and to identify variables associated with its occurrence. The secondary objective was to evaluate ultrasensitive troponin I as a predictor of hospital mortality.

After univariate analysis and multivariate logistic regression of a series of general variables and comorbidities, the predictors of myocardial injury with statistical significance were systemic arterial hypertension and body mass index. The mortality rate of this group was 24.6%, and the troponin I value was related to hospital mortality.

Although this study has important limitations, such as a small number of patients and loss of cases due to the absence of troponin I dosage, the impact of myocardial injury on in-hospital mortality and the identification of risk predictors such as systemic arterial hypertension and body mass index were demonstrated.

More robust, multicenter national studies may confirm the findings revealed in this study and draw a profile of the Brazilian population.
